# Synthesis, pharmacological evaluation, and *in silico* study of new 3-furan-1-thiophene-based chalcones as antibacterial and anticancer agents

**DOI:** 10.1016/j.heliyon.2024.e32257

**Published:** 2024-06-04

**Authors:** Ahmed Mutanabbi Abdula, Ghosoun Lafta Mohsen, Bilal H. Jasim, Majid S. Jabir, Abduljabbar I.R. Rushdi, Younis Baqi

**Affiliations:** a Department of Chemistry, College of Science, Mustansiriyah University, Baghdad, P.O. Box 14022, Iraq; b Department of Chemistry, College of Science, Nahrain University, Baghdad, P.O. Box 64074, Iraq; c Department of Applied Sciences, University of Technology, Baghdad, P.O. Box 19006, Iraq; d Department of Chemistry, College of Science, Sultan Qaboos University, Muscat, P.O. Box 36, Oman

**Keywords:** Antibacterial, Antioxidant, Breast cancer, Chalcone, Molecular docking

## Abstract

New 3-furan-1-thiophene-based chalcones were synthesized, characterized and pharmacologically evaluated as antibacterial and anticancer agents against two bacterial species; Gram-positive (*Streptococcus pyogenes*) and Gram-negative (*Pseudomonas aeruginosa*). All tested final compounds were active against the two bacterial species; *S. pyogenes* and *P.aeruginosa*. Especially compound AM4 showed large inhibition zone (27.13 and 23.30 mm), respectively. Using the DPPH assay, the new chalcones were evaluated for their free radical scavenging activity and found to reach up to 90 %, accomplished at a test concentration of 200 μg/mL. Furthermore, the chalcone derivatives were investigated against two breast cell lines; MCF-7 (cancerous) and MCF-10A (non-cancerous). Compound AM4 showed potent anticancer activity (IC_50_ = 19.354 μg/mL) in comparison to the other tested chalcone derivatives. *In silico* study was achieved using the PyRx AutoDock Vina software (0.8) to study the interaction types between the new hits and the binding sites of targeted proteins; glucosamine-6-phosphate synthase and tubulin, the target for antibacterial and anticancer drugs, respectively. Based on the molecular docking results the tested chalcones bind to the active pocket of the respective proteins, which support the *in vitro* results. In conclusion, 3-furan-1-thiophene-based chalcones could serve as new hits in the discovery of novel anticancer and/or antibacterial drugs.

## 1 Introduction

Cancer is the second most leading cause of death globally, with an estimation of one in every six deaths in 2020, which is nearly 10 million [1]. Breast cancer is the most frequent cancer with a total of 2.3 million new cases reported in 2020 [2,3]. The currently employed methods for breast cancer treatment include prophylactic mastectomy, chemotherapy, radiotherapy, medicines, and gene therapy [[4], [5], [6]]. However, these methods of treatments are usually associated with pain and considered expensive particularly in countries with low- and middle-income rates [7,8].

The reactive nitrogen/oxygen species (RNS/ROS) are compounds formed naturally as the byproducts of essential cellular processes [9,10]. Free radical scavengers, such as antioxidants controlling the presence of free radicals (ROS/RNS) in the cells [11,12]. A balance between antioxidants and free radicals is crucial for the normal physiological functions of the cells. If the percentage of free radicals exceeds the body's ability to control, oxidative stress will occur leading to several diseases such as cancer [13]. On the other hand, bacterial infectious diseases among the most serious problems worldwide due to antimicrobial resistance (AMR). As a result this may lead to morbidity and mortality [14]. Due to lack of new potent antimicrobial agents, several microbial strains that are previously appeared to be under control are now mutated to resist the known antibacterial agents, causing nearly 5 million deaths every year [15].

Natural products isolated from food, medicinal herbs, and their derived bioactive compounds have been reported to be less toxic compared with synthetic agents [[16], [17], [18], [19], [20]]. Therefore, natural products inspired small molecules might be a suitable alternatives for breast cancer therapy.

Chalcone moiety, 1,3-diaryl-propenone, is a key intermediate metabolite in the biosynthesis of flavonoids, a naturally occurring group of compounds. Chalcone skeleton comprises of two aryl/heteroaryl entities separated by three carbon atoms, generating an α,β-unsaturated carbonyl compound. Chalcone compounds have been well documented in the literature to exhibit several pharmacological activities such as anti-inflammatory [[21], [22], [23]], antiviral [24], anticancer [[25], [26], [27]], antibacterial [[28], [29], [30], [31]], and antioxidant [32,33].

In this study, new 3-furan-1-thiophene-based chalcones (AM1–AM4), were synthesized in good isolated yield and pharmacologically evaluated as antibacterial agents against *Streptococcus pyogenes* (Gram-positive) and *Pseudomonas aeruginosa* (Gram-negative), moreover, the target compounds (AM1–AM4) were investigated as antioxidant, cytotoxic, and apoptosis activities against two breast cell lines, cancerous MCF-7 (Michigan Cancer Foundation-7) and non-cancerous MCF-10A cell line.

**Fig. 1:**
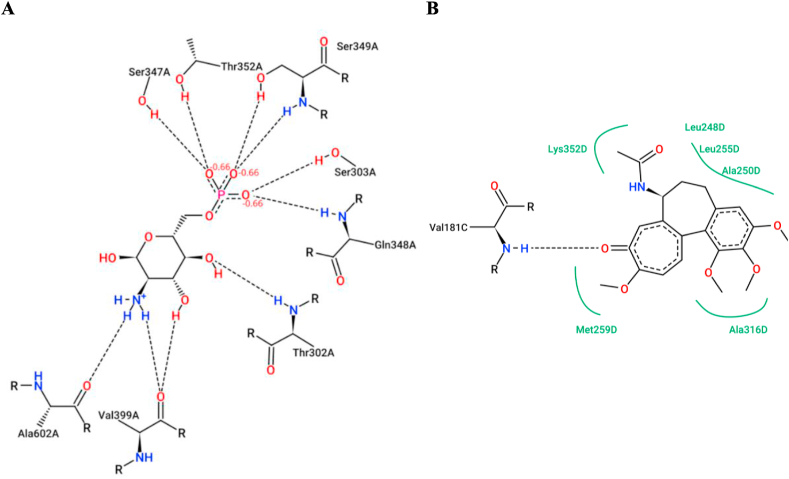
Two-dimensional diagrams highlighted the active sites of the studied proteins: **A.** The active site of the enzyme GlcN-6-P synthase showing the interactions between the natural ligand glucosamine-6-phosphate and amino acids; **B.** Tubulin (receptor) in complex with the drug (colchicine). (Obtained from Proteins Plus, a pose view website, at https://proteins.plus/(accessed on February 2, 2024)).

In order to investigate the binding affinity of these new 3-furan-1-thiophene-based chalcones against the respective proteins, *in silico* studies were performed on GlcN-6-P synthase (glucosamine-6-phosphate synthase), the target enzyme for antimicrobial drugs [34] and colchicine binding site in tubulin, are the target receptors for anticancer drugs [35]. GlcN-6-P synthase binding site involves eight amino acids, these are Thr302, Ser303, Ser347, Gln348, Ser349, Thr352, Val399, and Ala602, while the binding pocket of tubulin comprises Val181, Leu248, Ala250, Leu255, Met259, Ala316, and Lys352. The posies view of the active binding sites of the studied proteins, GlcN-6-P synthase and tubulin, are illustrated in Fig. 1A and B, respectively.

## 2 Results and discussion

### 2.1 Chemistry

**Scheme 1:**
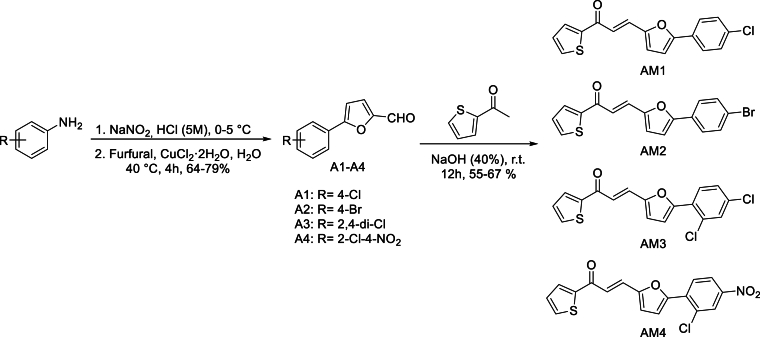
Synthesis of 5-aryl-2-furaldehyde intermediates (A1–A4) and their 3-furan-1-thiophene-based chalcones (AM1-AM4).

In the first reaction step, the intermediates, 5-aryl-furan-2-carboxaldehyde derivatives (A1–A4), were accessed in analogy to Meerwein synthetic protocol as previously described [18,36]. In brief, the aniline derivatives bearing electron withdrawing groups (4-Cl, 4-Br, 2,4-*di*-Cl, and 2-Cl-4-NO_2_) were diazotized using sodium nitrite (NaNO_2_) in the presence of 7 M hydrochloric acid (HCl) at 0–5 °C. To the prepared diazonium salt was added, without isolation, furfural, copper (II) chloride dihydrate (CuCl_2_·2H_2_O), and water. The resulting mixture was then warmed up to 40 °C for 4 h and the intermediates (A1–A4) were obtained in good isolated yields, see Scheme 1.

In the next step, 3-furan-1-thiophene-based chalcone derivatives (AM1–AM4) were synthesized using a modified Claisen-Schmidt condensation reaction [18,37]. An equimolar concentration of the 5-aryl-furan-2-carboxaldehyde derivatives (A1–A4), prepared in the previous step, and 2-acetylthiophene were mixed in the presence of aqueous NaOH (40 %) and ethanol. The obtain reaction mixture was stirred for 12 h at room temperature, furnishing the target compounds in moderate isolated yields (Scheme 1).

The structure elucidation of the new 3-furan-1-thiophene-based chalcones (AM1–AM4) were assessed by spectral data analysis, using FT-IR, ^1^H NMR, and GC-MS spectra, Figs. S1, S2, and S3, respectively, see Supporting Material. Since the structures of the synthesized chalcones are similar, except for the substituent attached to the phenyl ring, chalcone AM1 is taken to discuss for structure elucidation. The FT-IR spectrum of AM1 shows stretching absorption at around 3082 cm^−1^ for the aromatic C–H, while the C=O stretching absorption recorded at 1639 cm^−1^. The absorption peaks at 1579 and 1560 cm^−1^ are related to CH=CH chalcone and C=C aromatic. The ^1^H NMR of AM1 showed peaks for the thiophene, the phenyl ring, and the α,β-unsaturated carbonyl moieties. A two doublet signals at 7.21 and 7.27 ppm with *J* = 3.6 Hz related to two protons of the furan ring. The triplet signal at 7.34 (*J* = 4.4, 8.8 Hz) related to one proton of the thiophene moiety. The multiplet signal in the range 7.55–7.60 ppm related to one proton of CH=CHCO as well as two aromatic protons. The other CH=CHCO proton appear as doublet at 7.67 ppm with *J* = 15.2 Hz revealed the *trans* configuration of the double bond. Furthermore, the doublet signal at 7.99 ppm (*J* = 8.4 Hz) related to two aromatic protons. The two doublet signals at 8.08 and 8.32 ppm with coupling constant equal to 4.8 and 4.0 Hz, respectively, are related to the other thiophene protons. The structure of compound AM1 also confirmed via mass spectrometry analysis. The mass spectroscopy showed molecular ion peak at *m*/*z* = 315, which represent the molar mass of AM1 in the positive mode (M^+^). The other synthesized derivatives showed the spectral analysis (as depicted in the experimental section) according to the Claisen-Schmidt condensation between ketone and aldehydes used in this study furnishing chalcones (AM1–AM4).

### 2.2 Biology

#### 2.2.1 Antibacterial activity of the furan-thiophene-chalcone derivatives (AM1–AM4)

**Fig. 2:**
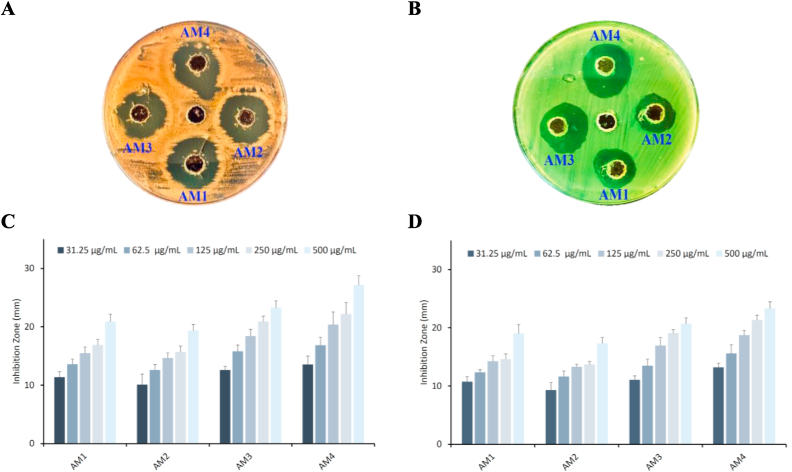
Antibacterial activity of furan-thiophene-chalcone derivatives (AM1–AM4) at different concentrations against *S. pyogenes* and *P. aeruginosa*. **A.** Inhibition zone *S. pyogenes*, **B.** Inhibition zone *P. aeruginosa*, **C.** Test compounds against *S. pyogenes*, and **D.** Test compounds against *P. aeruginosa*. Untreated bacterial strains were used as negative control. Results are reported as mean ± SD of three independent experiments.

The antibacterial activity of AM1, AM2, AM3, and AM4 were investigated against two human pathogenic bacteria; *S*. *pyogenes* (Gram-positive) and *P*. *aeruginosa* (Gram-negative). The antibacterial activity of the synthesized chalcones (AM1–AM4) are provided as inhibition zones in mm, see Fig. 2A and B. The antimicrobial results indicate that all the tested compounds showed significant antibacterial activity against both tested human pathogen bacteria in a concentration dependent manner, see Fig. 2C and D.

Chalcone AM4, bearing two electron withdrawing groups in the *ortho*- & *para*-position, Cl and NO_2_, respectively, showed the highest inhibition activity against both tested bacteria. Replacement of NO_2_ by a Cl group (AM3) showed the second most active compound, while the removal of the ortho functional group (AM1) slightly decreased the activity. Replacement of the *para*-Cl by a Br (AM2) resulted in decreasing the activity.

**Fig. 3:**
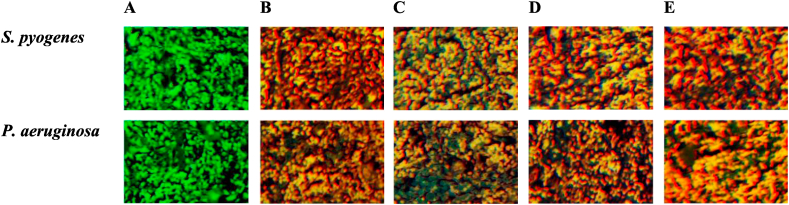
Live and died bacterial strains using AO/EtBr stain. **A.** Control, **B.** AM1, **C.** AM2, **D.** AM3, and **E.** AM4.

These results suggest that the presence of electron withdrawing functions in the *ortho*- and *para*-position is essential for the antibacterial activity of these kind of chalcone derivatives. Utilizing fluorescence microscopy, AM1–AM4 activity was assessed. In this experiment, living and dead bacterial strains treated with AM1–AM4 were distinguished using the acridine orange/ethidium bromide (AO/EtBr) double stain. Fluorescent green light is created when acridine orange binds to the nucleic acid of viable bacteria (*S. pyogenes* and *P. aeruginosa*) (Fig. 3A), ethidium bromide adsorbs on the nucleic acid of the dead bacteria, resulting in red or orange fluorescence (Fig. 3B–E).

The results suggests that the tested 3-furan-1-thiophene-based chalcones have preventive effects against *S. pyogenes* and *P. aeruginosa*. The antimicrobial effects is probably result from the interaction of the synthesized compounds with the target microorganism's cell membrane as well as from their ability to interact to cell walls, absorbable proteins, and outer cells [38].

#### 2.2.2 DPPH assay: Antioxidant activity of 3-furan-1-thiophene-based chalcones (AM1–AM4)

**Fig. 4:**
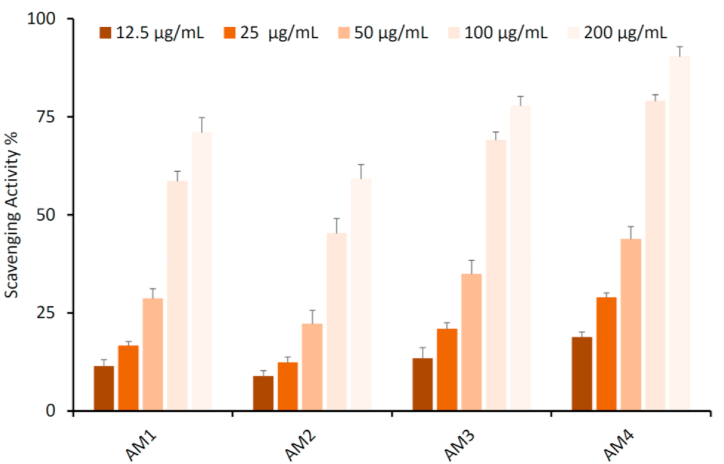
Antioxidant activity of furan-thiophene-chalcones (AM1–AM4). Results are reported as mean ± SD of three independent experiments.

The antioxidant activity of the newly synthesized chalcones (AM1–AM4) was accomplished using DPPH (2,2-diphenyl-1-picrylhydrazyl) assay. All the tested compounds showed a radical-scavenging percent activity in a concentration dependent manner as illustrated in Fig. 4. The type of substituent on the chalcone moiety play a significant role on the magnitude of the scavenging activity percent. A compound bearing strong electron withdrawing group (NO_2_) in the *para*-position as well as moderate electron withdrawing group (Cl) in *ortho*-position showed excellent antioxidant activity, up to 90 %, see AM4 (Fig. 4). The deceasing in the electron withdrawing properties lead to decrease in the scavenging activity, e.g., the presence of a Br group in the *para*-position lead to the least activity (up to 60 %) of the tested compounds, see AM2 (Fig. 4).

#### 2.2.3 LDH assay: Cytotoxicity analysis in breast cancer cells

**Fig. 5:**
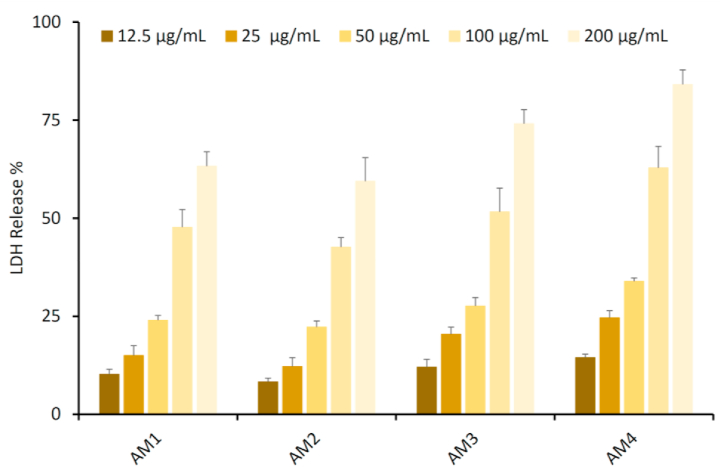
Furan-thiophene-chalcone derivatives (AM1–AM4) induces release of LDH in MCF-7 cell line. Results are reported as mean ± SD of three independent experiments.

Lactate dehydrogenase (LDH) is one of the enzymes that releases in the extracellular site when cells are damaged, which acts as a biomarker of cells undergoing apoptosis or cell lysis as an indication of cellular damage. The LDH concentration in the extracellular cite is used as a positive indication of cytotoxicity in cancer cells. The activity of the LDH enzyme was evaluated in the presence of the synthesized chalcone derivatives (AM1–AM4) using the following concentrations (12.5, 25, 50, 100, and 200 μg/mL) to study their growth inhibition ability against MCF-7 cancer cell line. All the tested compounds showed inhibition activity in a concentration dependent manner, Fig. 5. Among tested compounds, AM4 treated MCF-7 cells shows a significant release in LDH level at 200 μg/mL compared with their level at 12.5 μg/mL, indicating the cell lysis. The LDH assay suggest the potent activity of compound AM4 against the proliferation in the breast cancer cell line (MCF-7).

#### 2.2.4 MTT assay: Anticancer activity of 3-furan-1-thiophene-based chalcones (AM1–AM4)

**Fig. 6:**
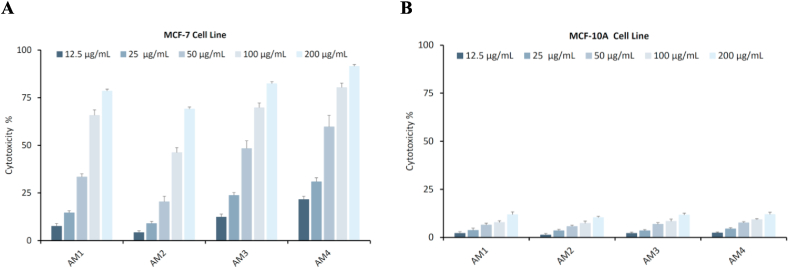
Cytotoxic activity of furan-thiophene-chalcone derivatives (AM1–AM4). **A.** MCF-7 cell line and **B.** MCF-10A cell line. Results are reported as mean ± SD of three independent experiments.

The anticancer activity of the AM1–AM4 were investigated at two human breast cell lines, MCF-7 (cancerous) and MCF-10A (non-cancerous), using the 3‐(4,5‐dimethyl‐2‐thiazolyl)‐2,5‐diphenyl‐2*H*‐tetrazolium bromide (MTT) assay. The percentage of viable MCF‐7 cells for the four investigated compounds (AM1–AM4) at the following concentrations (12.5, 25, 50, 100, and 200 μg/mL) is outlined in Fig. 6A. The cytotoxicity of all tested compounds found to be in a concentration-dependent manner. The consequences of the obtained IC_50_ concentration of chalcones AM1–AM4, that were deduced from the relationship between the compound concentrations and their cytotoxic effects on MCF‐7 cell viability, were 27.456, 25.870, 22.870, and 19.354 μg/mL, respectively. Compound AM4 shows the highest preventive effect on cell proliferation of MCF-7 than other tested compounds (AM1–AM3). In contrast, all tested compounds shows no toxicity against MCF-10A (Fig. 6B), which is the most common cell line used as a model for normal human breast cells. This result suggests that compound AM4, with its lower IC_50_ concentration (19.354 μg/mL), may have greater potential anticancer properties.

#### 2.2.5 Apoptosis assay of the synthesized compounds (AM1–AM4)

**Fig. 7:**
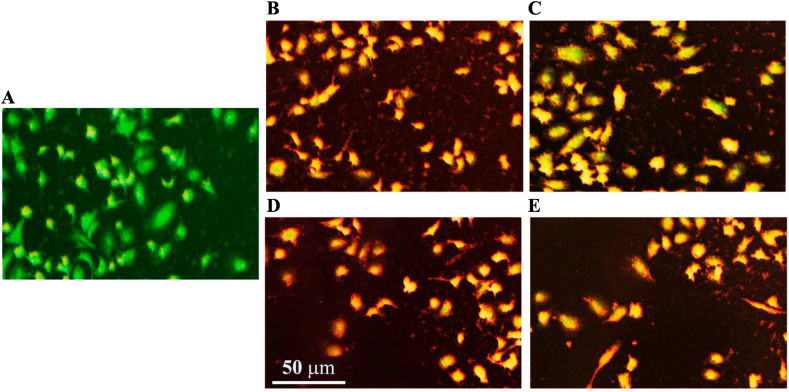
Apoptosis markers in MCF-7 cell line following treatment with furan-thiophene-chalcone derivatives (AM1–AM4). **A.** Control cells, **B.** AM1 treated cells, **C.** AM2 treated cells, **D.** AM3 treated cells, and **E.** AM4 treated cells. Test compounds concentration 200 μg/mL. Scale bar 50 μm.

The nuclear morphology changes were investigated after the treatment of the breast cancer cells (MCF-7) with the test compounds (AM1–AM4). The results are outlined in Fig. 7. Acridine orange/ethidium bromide (AO/EtBr) dual labeling stain was used to assess the nuclear morphology of the treated MCF-7 cells. DNA damage was used to analyze cells that undergo apoptosis. After staining with AO/EtBr, apoptotic cells showed an orange or red color (Fig. 7B–E), while non-apoptotic cells showed a green color (Fig. 7A) [39].

#### 2.2.6 Impacts of the synthesized compounds (AM1–AM4) on mitochondrial membrane potential

The potential of mitochondrial membrane is a significant indicator and possible target for the treatment of cancer cells, where apoptosis-inducing mitochondrial dysfunction is a prominent feature. As a result, the induction of the mitochondrial membrane depolarization by treatment with synthesized compounds (AM1–AM4) caused the integrity of the mitochondria to be destroyed. Mitochondria are essential for the stimulation of apoptotic processes. This organelle is altered when its membrane potential (Δψm) is lost and the cytochrome *c* protein is released into the cytoplasm, which causes caspase-3 to be upregulated via the caspase-9 pathway.

**Fig. 8:**
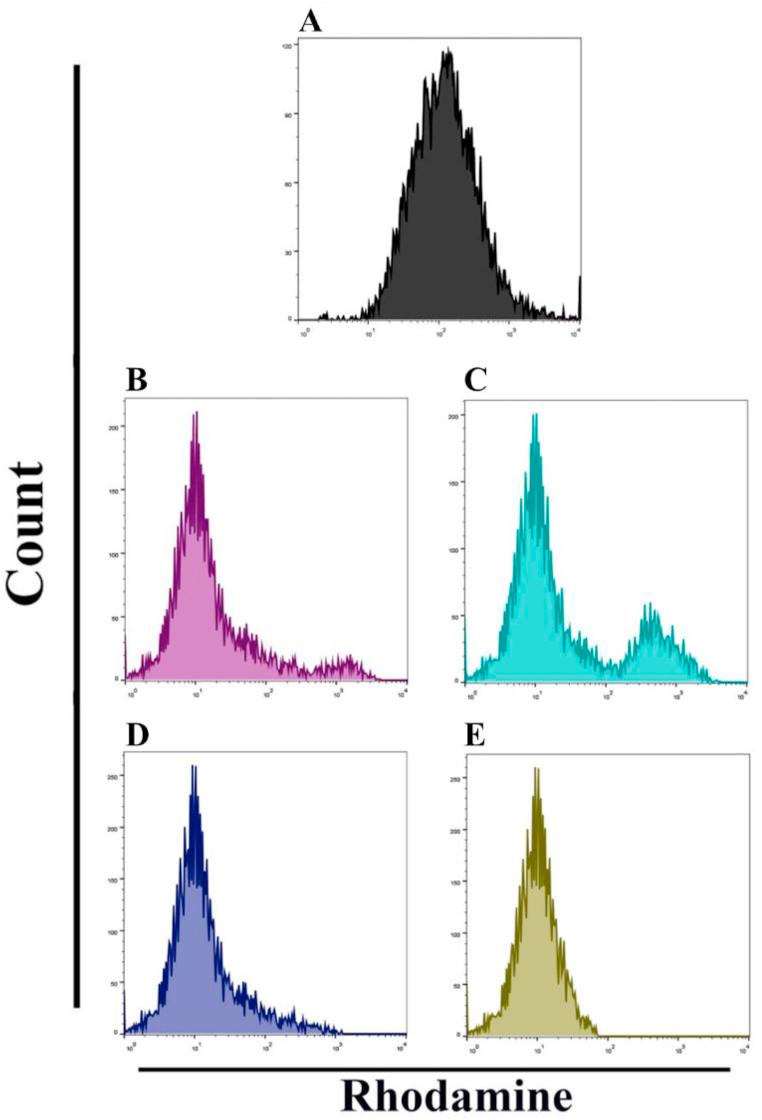
Dysfunction of MMP in MCF-7 cell line. Mitochondrial dysfunction was measured by Rhodamine stain. Flow cytometry data in cells treated with test compounds (AM1–AM4). **A.** Control cells, **B.** AM1 treated cells, **C.** AM2 treated cells, **D.** AM3 treated cells, and **E.** AM4 treated cells.

Following the manufacturer's instructions, we used a flow cytometry technique to identify apoptosis in the current investigation. One crucial biomarker of apoptotic cell death is a reduction in the potential of the mitochondrial membrane. Rh123 probe labeling of MCF-7 cells was followed by the measurement of the mitochondrial membrane potential using the flow cytometry technique. We investigated the number of MCF-7 cells that underwent apoptosis following the treatment with the test compounds (AM1–AM4). Fig. 8 illustrates the significant increase in apoptosis that was seen upon treatment with the tested compounds (AM1–AM4, Fig. 8B–E). When compared to the untreated control MCF-7 cells (Fig. 8A), MCF-7 cells treated with AM4 (Fig. 8E) for 24 h showed a significant decrease in Rh123 staining, indicating a reduction in the potential of the mitochondrial membrane. The activity of the synthesized chalcones (AM1–AM4) is proportionate to their affinity for the tubulin target's colchicine binding site, as indicated by the docking investigation (section 2.3).

### 2.3 *In silico* study

#### 2.3.1 Molecular docking of the synthesized compounds (AM1–AM4) to glucosamine-6-phosphate (GlcN-6-P) synthase

Glucosamine-6-phosphate synthase (GlcN-6-P synthase) is one of the targeted enzymes for antimicrobial chemotherapy. GlcN-6-P synthase plays crucial role in construction of bacterial cell wall through the biosynthesis of macromolecules bearing sugar moiety. The first reaction catalyzed by this enzyme is the formation of d-glucosamine-6-phosphate (GlcN-6-P) form d-fructose-6-phosphate (Fru-6-P) followed by the formation of uridine-5-diphospho-*N*-acetyl-d-glucosamine (UDP-GlcNAc), the important component for the cell wall assembly in bacteria [40].

**Fig. 9:**
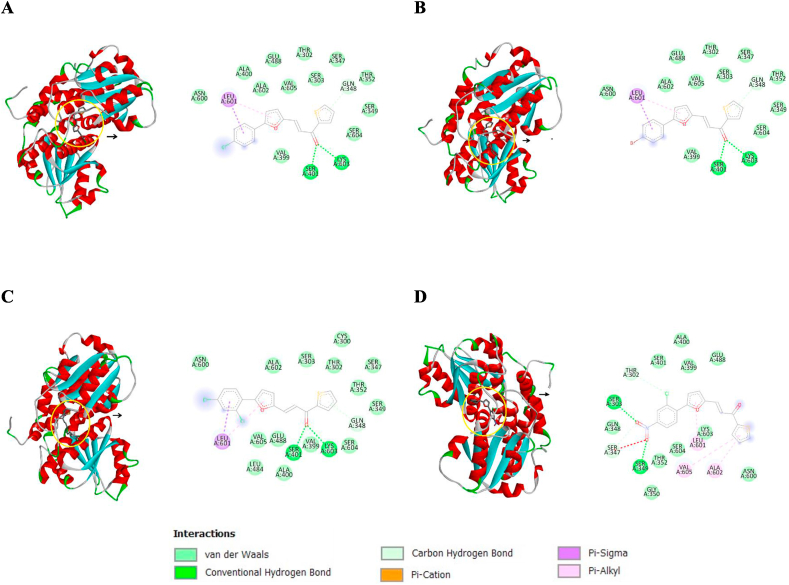
Binding of the furan-thiophene-chalcone derivatives (AM1–AM4) inside the GlcN-6-P synthase. **A.** AM1, **B.** AM2, **C.** AM3, and **D.** AM4.

The synthesized derivatives (AM1–AM4) were docked inside the active site of GlcN-6-P synthase (PDB: 1MOQ) after the removal of the substrate glucosamine-6-phosphate to examine the interaction mode and the binding affinity toward the enzyme. The docking outcomes indicate that compound AM4 exhibited the best binding energy equal to 7.8 kcal⋅mol^−1^. The other synthesized compounds (AM1–AM3) show similar binding energies of 6.9 kcal⋅mol^−1^. The interaction types for all synthesized compounds (AM1–AM4) within the binding pocket of the GlcN-6-P synthase are provided in Fig. 9A–D, respectively. Compound AM4 binds the active site with two hydrogen bonds with serine amino acids; SER303 and SER349 residue. Furthermore, compound AM4 showed two van der Waals interactions with THR302 and SER347 residues. Additionally the amino acid residues LEU601, ALA602, and VAL605 display three π alkyl interactions with AM4. The red dot line represents the unfavorable acceptor-acceptor interaction between AM4 and SER347 residue.

The docking results strongly confirm the antibacterial activity of the synthesized chalcone derivatives. The nitro group in AM4 probably played crucial role in the *in vitro* activity as supported by molecular docking results. The presence of the nitro group in the *para*-position of the phenyl moiety found to make two hydrogen bonds with two serine amino acids (SER303 and SER349) residues, which probably contributed to the *in vitro* activity.

The reproducibility and accuracy of this *in silico* protocol was validated as previously described [22].

#### 2.3.2 Molecular docking of the synthesized compounds (AM1–AM4) to tubulin

**Fig. 10:**
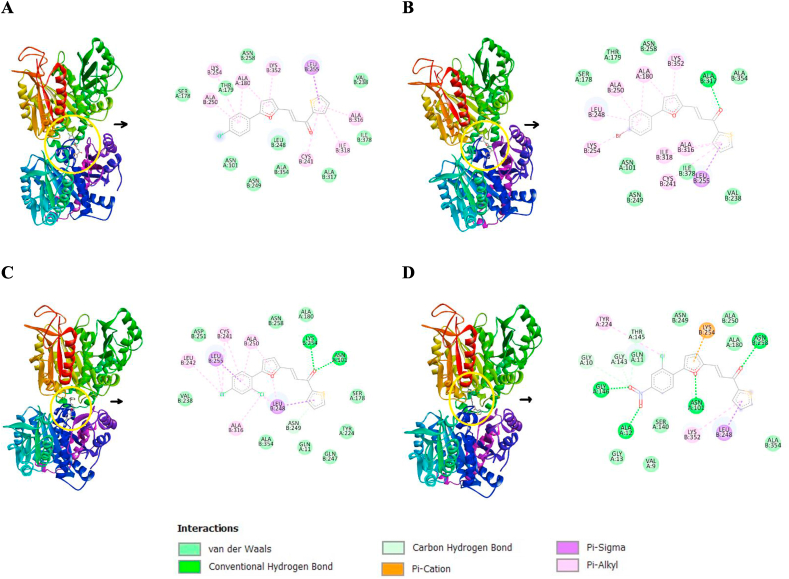
Binding of the furan-thiophene-chalcone derivatives (AM1–AM4) inside the tubulin receptor. **A.** AM1, **B.** AM2, **C.** AM3, and **D.** AM4.

Tubulin is a dimeric receptor comprises two subunits, α and β, that are related but not identical. Colchicine and its derivatives found to bind to tubulin and inhibit its polymerization, causing an abrupt disruption of mitotic spindle assembly, interference with the cytoskeleton's function, and interrupted mitosis, hence, contributed in blocking metastasis in various cancers [41]. The novel synthesized compounds (AM1–AM4) were *in silico* investigated against tubulin colchicine binding site (PDB: 4O2B) to study the action mechanism of these new skeleton as potential anticancer agents. The molecular docking was done to analyze the selectivity of the synthesized ligands based on their docking affinity. The docking score against tubulin showed that all the new ligands had potent affinity. The binding affinities of docked ligands AM1–AM4 were −8.2, −8.2, −8.5, and −9.2 kcal⋅mol^−1^, respectively. Compound AM4 showed the most potent *in silico* activity against the colchicine active site. It exhibits six types of interactions including van der Waals, hydrogen, carbon hydrogen, π cation, π sigma, and π alkyl bonds (Fig. 10D).

AM4 found to interact with the active site of the receptor through three van der Waals interactions with residue GLN:A11, GLY:A143 and GLY:A10 as well as three hydrogen bonds with the residue GLY:A146, ALA:A12 and ASN:B258. The furan moiety of AM4 binds the LYS:B254 with π cation bond. On the other hand, the thiophene ring interacts with beta site (LYS:B352 and LEU:B248) via π alkyl and π sigma bonds. The other π alkyl interaction was between the chlorine atom and the alpha residue TYR:A224.

The presence of nitro function in the *para*-position of the phenyl moiety probably contributed to the *in vitro* activity via performing these two hydrogen bonds with two amino acid, GLY:A146 and ALA:A12, residues. Furthermore, compounds AM1–AM3, Fig. 9A–C, respectively, also showed several interactions with active site as shown in Fig. 10. The docking results strongly confirm the anticancer activity of the synthesized chalcone derivatives.

The reproducibility and accuracy of this *in silico* method was validated. In brief, the co-crystalized drug (colchicine) was removed from its binding site within the target receptor (tubulin). In order to check the generated model, the ligand (colchicine) was redocked into the same active site of the receptor. The drug (colchicine) interacted with the generated model and fit back to its original site of the receptor.

## 3 Materials and methods

### 3.1 Chemistry

#### 3.1.1 General

All solvents and organic compounds were purchased from commercial suppliers (Sigma Aldrich, Merck, and BDH) and used as such without purification. The melting ranges of the new 3-furan-1-thiophene-based chalcones were determined using an electro thermal capillary apparatus (Digital Stuart scientific SMP30) and were uncorrected. The FT-IR spectrum was obtained using PLATINUM-ATR ALPHA II Spectrometer from Bruker Company. Gas chromatography-mass spectrum (GC-MS) was measured on GC-MS-QP2010 PLUS Shimadzu model. Nuclear magnetic resonance spectra (NMR) of the synthesized 3-furan-1-thiophene-based chalcones were collected on Bruker Avance Neo NMR spectrometer (400 MHz). The standard solvent peak appeared at *δ* 2.5 ppm related to DMSO‑*d*_6_.

#### 3.1.2 Synthesis of 5-aryl-furan-2-carboxaldehyde intermediates (A1–A4)

The 5-aryl-furan-2-carboxaldehydes (A1–A4) were synthesized and characterized following previously published modified procedure by our group [22,42]. In brief, aniline derivatives (0.136 mol) were diazotized with sodium nitrite NaNO_2_ (9.5 g, 0.138 mol) in the presence of hydrochloric acid (56.2 mL, 7 M). The obtained reaction mixture was stirred for 10 min and then faltered off. To the filtrate was added a solution of furan-2-carboxaldehyde (15.4 g, 0.160 mol) in water (50 mL), and cupric chloride dihydrate (5.0 g, 0.03 mol) dissolved in water (25 mL) at 10–15 °C. The reaction mixture let to stir at 40 °C for 4 h furnishing the target intermediates (A1–A4) in good isolated yields. The products were compared with authenticated samples obtained from our previously published work [22,42].

#### 3.1.3 Synthesis of 3-furan-1-thiophene-based chalcones (AM1–AM4)

Four new chalcone derivatives (AM1–AM4) were prepared via Claisen-Schmidt condensation reaction following previously published procedure [43,44]. A stirred solution of 2-acetyl thiophene (1 mmol) in ethanol (10 mL) was mixed with 40 % aqueous sodium hydroxide (1 mL). After stirring the mixture at room temperature for half an hour, 5-aryl-furan-2-carboxaldehyde derivatives (A1–A4), prepared in previous step, were added at an equimolar concentration (1 mmol) and the stirring kept for an additional 12 h. Upon reaction completion, followed by thin layer chromatography (TLC) using ethyl acetate/hexane (1:2) as the eluent, ice-cold water was added and the precipitate was filtered, washed with water (3 × 10 mL), allowed to air dry, and then recrystallized from ethanol. The stereoisomer of the chalcone double-bond generated in the final compounds (AM1–AM4) can easily be confirmed by the coupling constant (*J* values) about 15–16 Hz, of the vinylic protons, which indicate the *trans* (*E*) configuration of the double bonds. The structures of the final compounds were elucidated and confirmed by color, m. p., FT-IR, NMR, and GC-MS.

#### 3.1.4 Spectral data of the synthesized compounds (AM1–AM4)

(*E*)-3-[5-(4-Chloro-phenyl)-furan-2-yl]-1-thiophen-2-yl-propenone (AM1).

Yellow solid, yield 58 %, m. p. 155–157 °C; FT-IR (cm^−1^): 3082 (C–H aromatic), 2918 (C–H aliphatic), 1639 (C=O chalcone), 1579 (CH=CH chalcone), 1560 (C=C aromatic), 1029 (C–Cl). ^1^H NMR (400 MHz, DMSO‑*d*_6_) *δ* (ppm): 7.21 (d, 1H, CH furan, *J* = 3.6 Hz), 7.27 (d, 1H, CH furan, *J* = 3.6 Hz), 7.34 (t, 1H, CH thiophene, *J* = 4.4, 8.8 Hz), 7.55–7.60 (m, 3H, 2 Ar–H, CH chalcone), 7.67 (d, 1H, CH chalcone, *J* = 15.2 Hz), 7.99 (d, 2H, Ar–H, *J* = 8.4 Hz), 8.08 (d, 1H, CH thiophene, *J* = 4.8 Hz), 8.32 (d, 1H, CH thiophene, *J* = 4.0 Hz). GC-MS (EI) *m*/*z*: Calcd for C_17_H_11_ClO_2_S ([M]) 314.79; found 315 M^+^.

(*E*)-3-[5-(4-Bromo-phenyl)-furan-2-yl]-1-thiophen-2-yl-propenone (AM2).

Yellow solid, yield 55 %, m. p. 161–163 °C; FT-IR (cm^−1^): 3083 (C–H aromatic), 1636 (C=O chalcone), 1598 (CH=CH chalcone), 1576 (C=C aromatic), 1027 (C–Br). ^1^H NMR (400 MHz, DMSO‑*d*_6_) *δ* (ppm): 7.21 (d, 1H, CH furan, *J* = 3.6 Hz), 7.28 (d, 1H, CH furan, *J* = 3.6 Hz), 7.34 (t, 1H, CH thiophene, *J* = 3.6, 8.4 Hz), 7.57 (d, 1H, CH chalcone, *J* = 15.6 Hz), 7.65–7.71 (m, 3H, 2 Ar–H, CH chalcone), 7.92 (d, 2H, Ar–H, *J* = 8.4 Hz), 8.08 (d, 1H, CH thiophene, *J* = 3.6 Hz), 8.32 (d, 1H, CH thiophene, *J* = 4.0 Hz). GC-MS (EI) *m*/*z*: Calcd for C_17_H_11_BrO_2_S ([M]) 359.24; found 359 M^+^.

(*E*)-3-[5-(2,4-Dichloro-phenyl)-furan-2-yl]-1-thiophen-2-yl-propenone (AM3).

Orange solid, yield 62 %, m. p. 148–150 °C; FT-IR (cm^−1^): 3099 (C–H aromatic), 1650 (C=O chalcone), 1595 (CH=CH chalcone), 1562 (C=C aromatic), 1031 (C–Cl). ^1^H NMR (400 MHz, DMSO‑*d*_6_) *δ* (ppm): 7.24 (d, 1H, CH furan, *J* = 3.6 Hz), 7.32 (t, 1H, CH thiophene, *J* = 4.0, 8.8 Hz), 7.38 (d, 1H, CH furan, *J* = 3.6 Hz), 7.56–7.60 (m, 2H, 1 Ar–H, CH chalcone), 7.69 (d, 1H, CH chalcone, *J* = 15.2 Hz), 7.77 (s, 1H, Ar–H), 8.08 (d, 1H, CH thiophene, *J* = 4.0 Hz), 8.20 (d, 1H, Ar–H, *J* = 8.8 Hz), 8.29 (d, 1H, CH thiophene, *J* = 4.0 Hz). GC-MS (EI) *m*/*z*: Calcd for C_17_H_10_Cl_2_O_2_S ([M]) 349.23; found 349 M^+^.

(*E*)-3-[5-(2-Chloro-4-nitro-phenyl)-furan-2-yl]-1-thiophen-2-yl-propenone (AM4).

Orange solid, yield 67 %, m. p. 191–193 °C; FT-IR (cm^−1^): 3104 (C–H aromatic), 1649 (C=O chalcone), 1598 (CH=CH chalcone), 1581 (C=C aromatic), 1508 asym, 1339 sym (NO_2_), 1031 (C–Cl). ^1^H NMR (400 MHz, DMSO‑*d*_6_) *δ* (ppm): 7.30–7.34 (m, 2H, CH furan, CH thiophene), 7.60 (d, 1H, CH chalcone, *J* = 15.6 Hz), 7.63 (d, 1H, CH furan, *J* = 4.0 Hz), 7.74 (d, 1H, CH chalcone, *J* = 15.2 Hz), 8.08 (d, 1H, CH thiophene, *J* = 4.8 Hz), 8.24–8.29 (m, 2H, Ar–H), 8.38 (d, 1H, CH thiophene, *J* = 2.8 Hz), 8.44 (d, 1H, Ar–H, *J* = 8.8 Hz). GC-MS (EI) *m*/*z*: Calcd for C_17_H_10_ClNO_4_S ([M]) 359.78; found 360 M^+^.

### 3.2 Biology

#### 3.2.1 Antibacterial activity of furan-thiophene-chalcone derivatives (AM1–AM4)

The antibacterial properties of the synthesized chalcones (AM1–AM4) were tested against *S. pyogenes* (Gram-positive) and *P. aeruginosa* (Gram-negative) bacteria using agar well diffusion assay. Bacterial strains were gift from Biotechnology division, University of Technology, Baghdad, Iraq. In brief, Müller–Hinton agar (3.8 g) were dissolved in deionized water (100 mL). The resulting medium mixture was placed in an autoclave. The sterilization cycle was performed according to the Indian Pharmacopoeia. In the next step, nutrient agar (20 mL) was placed in sterilized Petri dishes. A sterile loop was used to take bacterial strains from stock bacterial cultures. Bacterial inoculums were spread on Müller–Hinton agar. Using a sterile tip, wells (6 mm diameter) were holed in the agar medium. Test compounds at concentration 31.25, 62.5, 125, 250 and 500 μg/mL in DMSO were placed in the wells. In the next step the plates comprises the test compounds and respective microorganism were incubated for 24 h at 37 °C. The antibacterial potencies of test compounds were assisted as zones of inhibition.

The inhibition zones were measured in mm, using a ruler and taking in consideration the average value of each well. The antibacterial activity tests were performed in triplicate and acridine orange/ethidium bromide (AO/EtBr) (Sigma Aldrich, USA) double stain was used to detect live and die bacterial stains [39].

#### 3.2.2 Antioxidant activity of chalcone derivatives (AM1–AM4) using DPPH assay

The antioxidant activity of the new 3-furan-1-thiophene-based chalcones (AM1–AM4) were measured using the free radical scavenger 2,2-diphenylpicrylhydrazyl (DPPH) assay (Sigma Aldrich, USA, 257,621) [45]. In brief, DPPH (500 μL) was combined with the chalcone derivatives (500 μL) at the following concentrations (12.5, 25, 50, 100, and 200 μg/mL), and the resulting volume was increased to 2 mL by adding absolute ethanol and the absorbance was recorded at 517 nm using UV–VIS spectrophotometer.

#### 3.2.3 LDH release assay

Lactate dehydrogenase (LDH) assay was performed following published procedure [46]. The media used to culture breast cancer cell line (MCF-7) did not contain phenol red indicator. The chalcone compounds (AM1–AM4) were then added to the cells at the following concentrations (12.5, 25, 50, 100, and 200 μg/mL) for 24 h. As a positive control, triton-X was employed. For 2 h, samples were incubated for 24 h at 37 °C. When the incubation period is over, all test and control wells (75 μL) aliquots were transferred into a 96-well plate. In the next step, each sample received 50 μL of the CytoTox 96VR Reagent (Sigma Aldrich, USA, MAK529). To shield it from light, the plate was wrapped in foil and incubated at room temperature for 30 min. Then the stop solution (50 μL) was added and kept for extra 30 min, and the absorbance was recorded at 490 nm.

#### 3.2.4 Cytotoxicity (MTT) assay

The cytotoxicity of the final compounds (AM1–AM4) were investigated using 3‐(4,5‐dimethyl‐2‐thiazolyl)‐2,5‐diphenyl‐2*H*‐tetrazolium bromide (MTT) assay [47]. The assay performed in 96-well plates on two breast cell lines (MCF-7 and MCF-10A). Both cell lines were obtained from Iraqi center for cancer research and medical genetics, Mustansiriyah University, Baghdad, Iraq. 1 × 10^4^ cells/mL of these cells were planted per well, and the cells were grown for 24 h. After removal of the growing medium, 200 μL of fresh medium with the following doses (12.5, 25, 50, 100, and 200 μg/mL) of test compounds was added, incubation was performed over 48 h. The cells were treated with 2 mg/mL MTT solution (Invitrogen, Carlsbad, CA) for 3 h after being rinsed with PBS. The solution was removed, followed by the addition of 100 μL of DMSO (Scharlau, Spain, SU01511000) to each well. Using a microplate reader, the absorbance of the test compound was recorded at 492 nm.

#### 3.2.5 Viability test using acridine orange/ethidium bromide (AO/EtBr) staining assay

The appropriate number of MCF-7 cells were cultivated at 37 °C and test compounds (AM1–AM4) was added at the following doses (12.5, 25, 50, 100, and 200 μg/mL) for 24 h. After adding 10 μL of AO/EtBr dye to the cells, fluorescence microscopy was used to analyze the outcomes. The cells ability to absorb dye was used to measure the viability. The double-stranded DNA of only living and early apoptotic cells is bound by the AO dye, whereas the EtBrdye only binds to necrotic and dead cells [48].

#### 3.2.6 Mitochondrial membrane potential assay

Using the fluorescent dye Rhodamine 123 (Rh123) (Sigma-Aldrich, USA, 83,702), the effects of chalcones (AM1–AM4) on the mitochondrial activity of the breast cancer cell line (MCF-7) were examined. Using this dye, the mitochondria membrane potential was investigated both before and after the addition of the test compounds. In summary, the cells in 96-well plates were seeded, treated with chalcone compounds (AM1–AM4) at five different concentrations (12.5, 25, 50, 100, and 200 μg/mL). Then stained with Rh123 dye (10 mM) for 2 h at 37 °C and incubated for 24 h. After that, the cells were separated with 0.2 mL of 5 % trypsin-EDTA and centrifuged for 5 min at 300 rpm. Consequently, the cells were resuspended in FACS buffer, assessed using a flow cytometry test, and generated histograms [49].

### 3.3 *In silico* study

The crystal structure of d-glucosamine-6-phosphate (GlcN-6-P) in complex with GlcN-6-P synthase (PDB code 1MOQ) and colchicine in complex with tubulin (PDB code: 4O2B) were obtained from RCSB PDB at https://www.rcsb.org/(accessed on February 2, 2024). The water molecules were removed from the target proteins, while corrupted amino acids were repaired in the respected proteins. The crystal structure of the target enzyme (GlcN-6-P synthase) and receptor (tubulin) was processed by the PyRx AutoDock Vina software (0.8). Three-dimensional structures of the 3-furan-1-thiophene-based chalcones (AM1–AM4) were constructed as mol files using Chemdraw Ultra (7.0) and with the help of Open Babel program (3.1.1) were converted into PDB format files. The synthesized chalcone derivatives (AM1–AM4) were docked to the binding site after removal of the crystal structure of the substrate (GlcN-6-P) and the drug (colchicine) from GlcN-6-P synthase and tubulin, respectively. The determined dimension was X = 31.7, Y = 16.3, Z = −2.1 with size equal to 19.6, 22.4, and 21.1 for the GlcN-6-P synthase, while the dimension applied for the tubulin target was X = 18.7, Y = 64.2, Z = 44.2 with size equal to 24.9, 27.2, and 27.9 as the grid spacing and the affinity (kcal⋅mol^−1^) value was calculated. The best obtained Auto-Dock score and the optimal interactions for the interpretation of the best conformation was used [50,51]. The *in-silico* results were visualized and analyzed using Discovery studio 2021. The validity of docking protocols confirmed via redocking of crystalized substrate (GlcN-6-P) and drug (colchicine) within the binding pocket of the respective protein.

## 4 Conclusions

New 3-furan-1-thiophene-based chalcones (AM1–AM4) were synthesized, chemically characterized, and biologically evaluated as antibacterial and anticancer agents. The 3-furan-1-thiophene-chalcones were synthesized from the reaction between 2-acetylthiophene and the newly synthesized 5-aryl-2-furaldehyde intermediates (A1–A4). All synthesized products showed activity on the two investigated bacterial species, these are *S. pyogenes* (Gram-positive) and *P. aeruginosa* (Gram-negative). The synthesized compounds exhibited excellent antioxidant activity in the DPPH assay. Furthermore, the chalcone derivatives showed promising potency against MCF-7, the cancerous human breast cell line, with no toxicity against MCF-10A, the non-cancerous human breast cell line. *In silico* docking investigation was performed to explore the virtual affinity and the binding mode of the new compounds (AM1–AM4) inside the glucosamine-6-phosphate binding site of GlcN-6-P synthase as well as the colchicine binding site of tubulin. The docking investigations revealed that the tested compounds bind to the same active site of the substrate (GlcN-6-P) and drug (colchicine). The newly synthesized chalcone derivatives may serve as new hits for the discovery of novel antibacterial and/or anticancer agents. In the future work more derivatives need to be synthesized in order to explore the structure-activity relationships (SARs) of the 3-furan-1-thiophene-based chalcones in more details.

## Funding

This research was funded by the 10.13039/100019465Arab-German Young Academy of Sciences and Humanities (AGYA) grants (01DL20003).

## Institutional review board statement

Not applicable.

## Informed consent statement

Not applicable.

## Data availability statement

All data are provided in the published paper.

## CRediT authorship contribution statement

**Ahmed Mutanabbi Abdula:** Writing – original draft, Validation, Software, Resources, Methodology, Investigation, Formal analysis, Conceptualization. **Ghosoun Lafta Mohsen:** Investigation, Data curation. **Bilal H. Jasim:** Investigation, Formal analysis, Data curation. **Majid S. Jabir:** Writing – original draft, Resources, Methodology, Formal analysis, Conceptualization. **Abduljabbar I.R. Rushdi:** Formal analysis, Data curation. **Younis Baqi:** Writing – review & editing, Writing – original draft, Supervision, Project administration, Methodology, Funding acquisition, Conceptualization.

## Declaration of competing interest

The authors declare that they have no known competing financial interests or personal relationships that could have appeared to influence the work reported in this paper.
