# Supplementary material for: Synthesis, pharmacological evaluation, and in silico study of new 3-furan-1-thiophene-based chalcones as antibacterial and anticancer agents
Source: Heliyon. 2024 Jun 4;10(11):e32257. doi: 10.1016/j.heliyon.2024.e32257 (PMC11214363; doi:10.1016/j.heliyon.2024.e32257)
Supplement: Multimedia component 1 [file mmc1.docx]

Supplementary Material

for

Synthesis, Biological Evaluation, and In Silico Study of New 3-Furan-1-Thiophene-Based Chalcones as Antibacterial and Anticancer Agents

Ahmed Mutanabbi Abdula^1,^*, Ghosoun Lafta Mohsen^2^, Bilal H. Jasim^3^, Majid S. Jabir^3^, Abduljabbar I.R. Rushdi^1^ and Younis Baqi^4,^*

^1^Department of Chemistry, College of Science, Mustansiriyah University, Baghdad P.O. Box 14022, Iraq; ^2^Department of Chemistry, College of Science, Nahrain University, Baghdad P.O. Box 64074, Iraq; ^3^Department of Applied Sciences, University of Technology, Baghdad P.O. Box 19006, Iraq; ^4^Department of Chemistry, College of Science, Sultan Qaboos University, Muscat P.O. Box 36, Oman.

*****Correspondence: ahm.chem@uomustansiriyah.edu.iq; Tel.: +964-7808838128 (A.M.A.); baqi@squ.edu.om; Tel.: +968-2414-1473 (Y.B.)

**Table of contents**

| Contents | Page |
| --- | --- |
| Figure S1. FT-IR spectra of compounds AM1–AM4. | S2 |
| Figure S2. ^1^H-NMR spectrum of compounds AM1–AM4 in DMSO-*d*_6_. | S3 |
| Figure S3. GC-MS spectra of compounds AM1–AM4. | S4 |

| **A** | **B** |
| --- | --- |
| 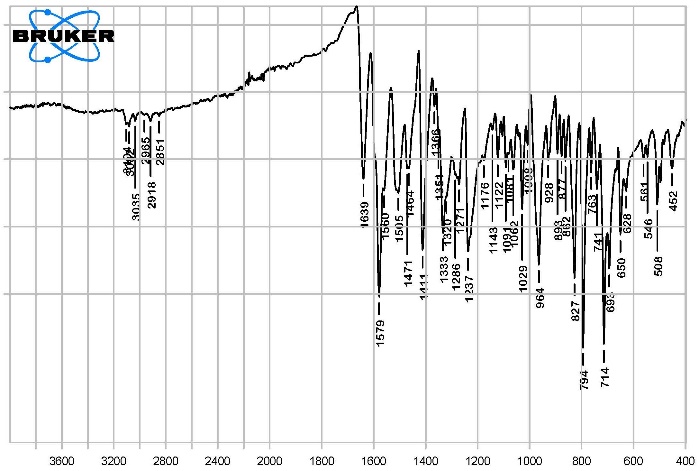 | 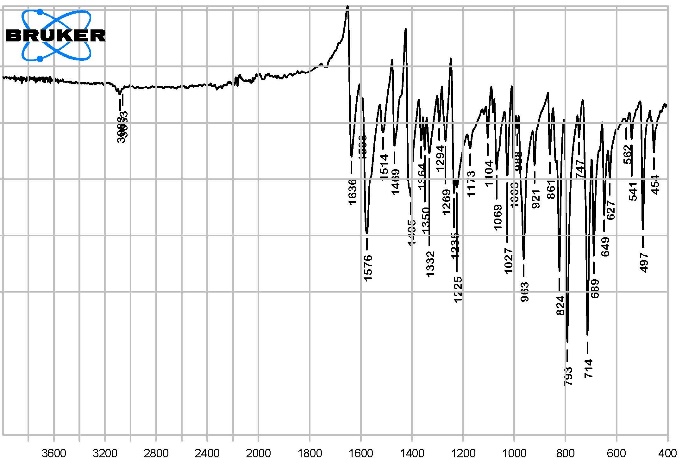 |
| **C** | **D** |
| 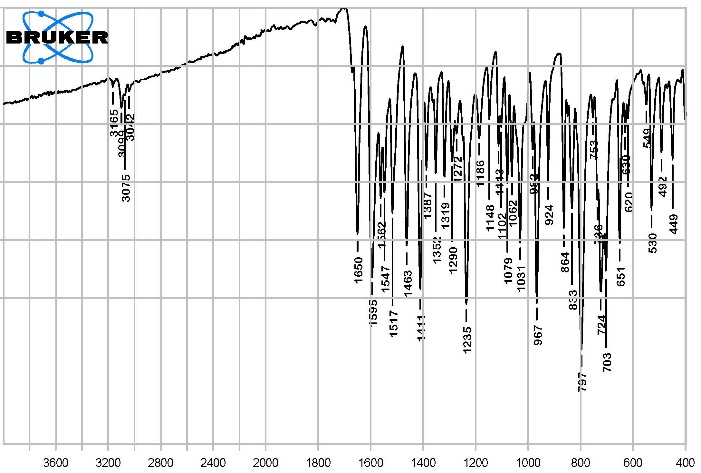 | 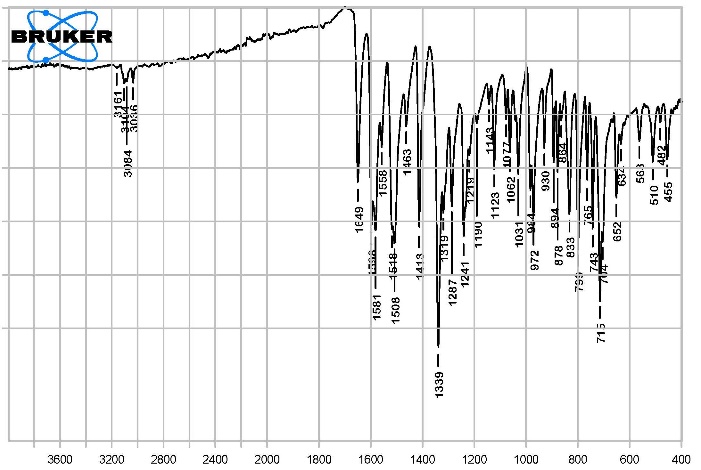 |

**Figure S1.** FT-IR spectra of furan-thiophene-chalcone derivatives; **A.** AM1, **B.** AM2, **C.** AM3, and **D.** AM4.

| **A** | **B** |
| --- | --- |
| 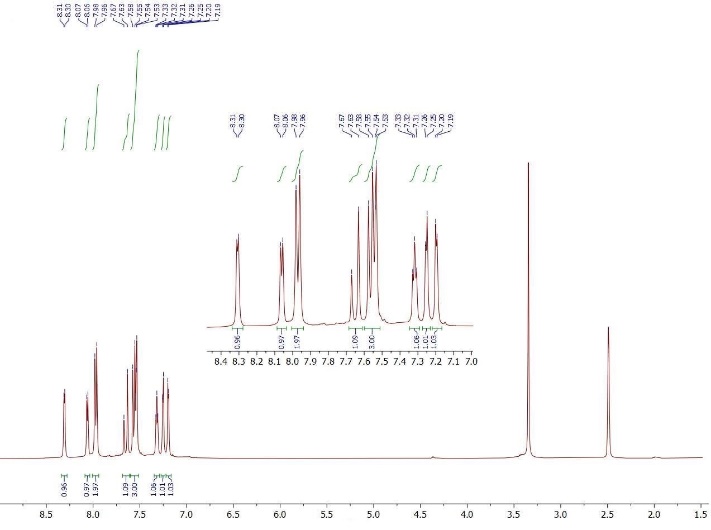 | 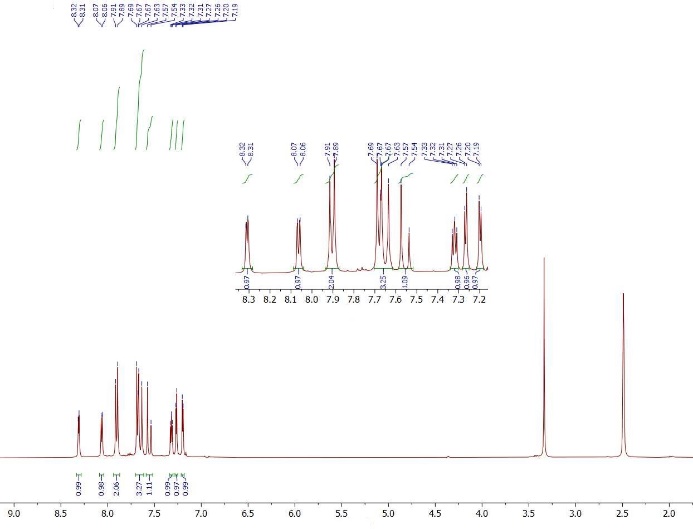 |
| **C** | **D** |
| 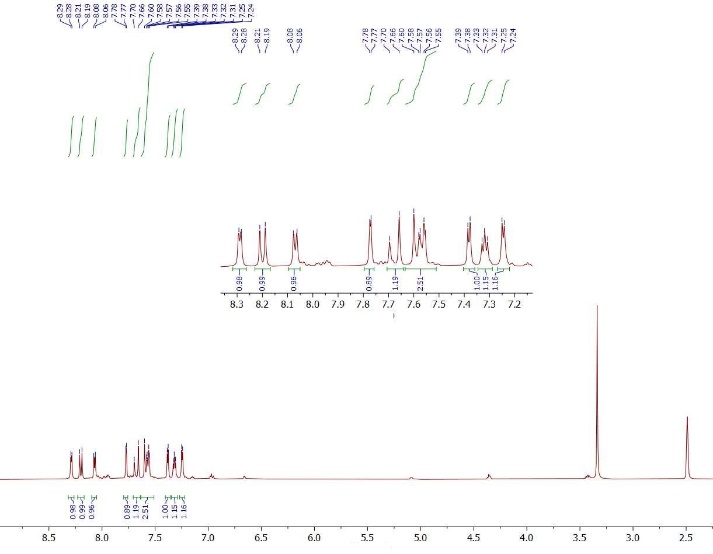 | 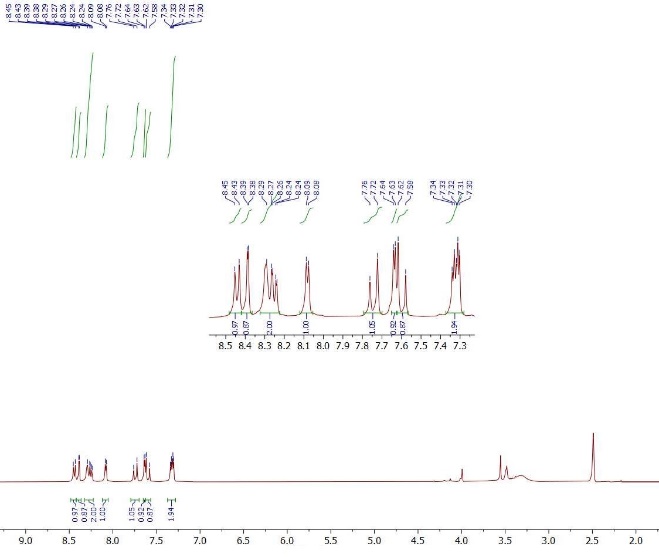 |

**Figure S2.** 1H-NMR spectra of furan-thiophene-chalcone derivatives; **A.** AM1, **B.** AM2, **C.** AM3, and **D.** AM4.

| **A** | **B** |
| --- | --- |
| 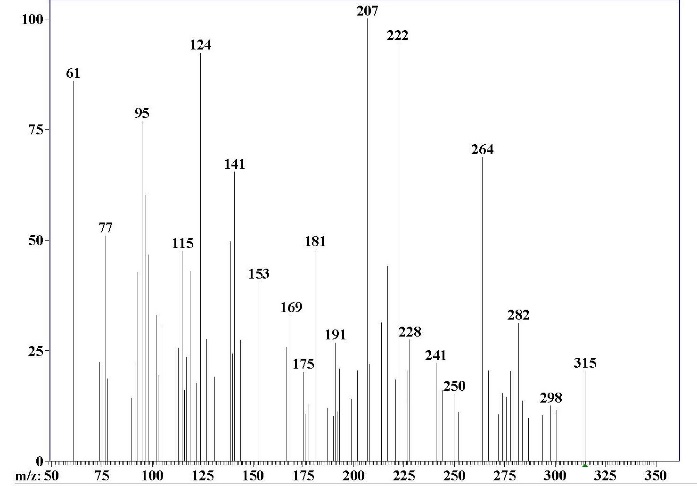 | 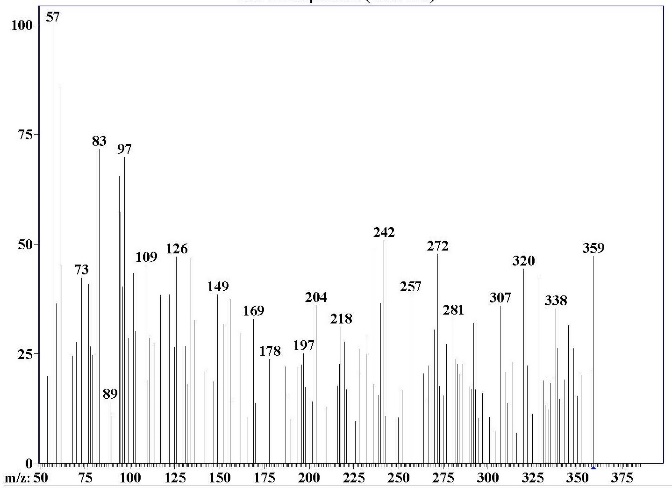 |
| **C** | **D** |
| 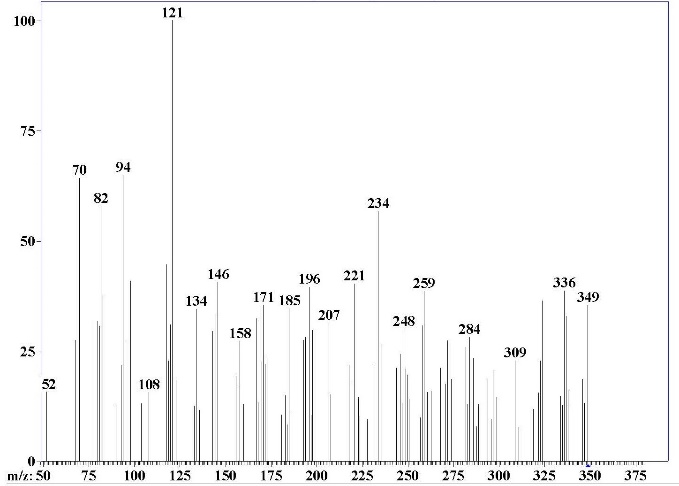 | 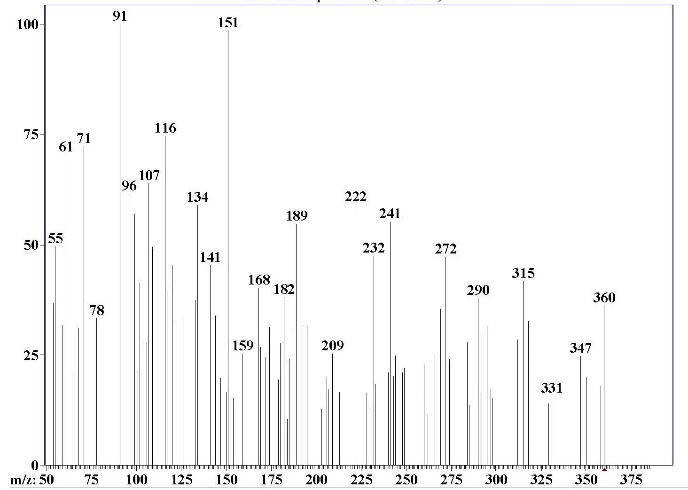 |

**Figure S3.** GC-MS spectra of furan-thiophene-chalcone derivatives; **A.** AM1, **B.** AM2, **C.** AM3, and **D.** AM4.
